# Supplementary material for: Polyketide synthase gene clusters in the ericoid mycorrhizal fungus Oidiodendron maius: genome-wide prediction, expression profiling, and functional characterization of knockout mutants
Source: Front Plant Sci. 2026 Sep 16;17:1924430. doi: 10.3389/fpls.2026.1924430 (PMC13623903; doi:10.3389/fpls.2026.1924430)
Supplement: Supplementary file 1 [file SupplementaryFile1.zip › Supplementary Materials/Supplementary Folder 1.zip!/Oidma1_AssemblyScaffolds_Repeatmasked/knownclusterblast/cluster54/jgi.p_Oidma1_96286_mibig_hits.html]

| MIBiG Protein | Description | MIBiG Cluster | MiBiG Product | % ID | % Coverage | BLAST Score | E-value |
| --- | --- | --- | --- | --- | --- | --- | --- |
| EHA28243.1 | hypothetical\_protein | BGC0001143 | Azanigerone\_biosynthetic\_gene\_cluster | 35 | 94.9044585987 | 280 | 3.4e-75 |
| EHA28234.1 | FAD/FMN-containing\_dehydrogenase | BGC0001143 | Azanigerone\_biosynthetic\_gene\_cluster | 32 | 94.0552016985 | 252 | 1.3e-66 |
| EAA65603.1 | hypothetical\_protein | BGC0000022 | Asperfuranone\_biosynthetic\_gene\_cluster | 32 | 96.6029723992 | 233 | 6.3e-61 |
| EAL89048.1 | FAD\_binding\_domain\_protein | BGC0000355 | Fumiquinazolines\_biosynthetic\_gene\_cluster | 30 | 101.486199575 | 216 | 8e-56 |
| ADY16695.1 | TqaG | BGC0001142 | Tryptoquialanine\_biosynthetic\_gene\_cluster | 28 | 98.5138004246 | 204 | 3.1e-52 |
| ABP55175.1 | FAD\_linked\_oxidase\_domain\_protein | BGC0000150 | Sporolide\_biosynthetic\_gene\_cluster | 29 | 57.5371549894 | 94 | 4.6e-19 |
| AAD32736.1 | MmcM | BGC0000915 | Mitomycin\_biosynthetic\_gene\_cluster | 29 | 36.093418259 | 83 | 8.1e-16 |
